# Reduction of Fusarium head blight and trichothecene contamination in transgenic wheat expressing *Fusarium graminearum* trichothecene 3-*O*-acetyltransferase

**DOI:** 10.3389/fpls.2024.1389605

**Published:** 2024-04-08

**Authors:** Gabdiel Yulfo-Soto, Susan McCormick, Hui Chen, Guihua Bai, Harold N. Trick, Guixia Hao

**Affiliations:** ^1^ Mycotoxin Prevention and Applied Microbiology Research Unit, National Center for Agricultural Utilization Research, Agricultural Research Service, USDA, Peoria, IL, United States; ^2^ Oak Ridge Institute for Science and Education, Mycotoxin Prevention and Applied Microbiology Research Unit, National Center for Agricultural Utilization Research, Agricultural Research Service, USDA, Peoria, IL, United States; ^3^ Department of Agronomy, Kansas State University, Manhattan, KS, United States; ^4^ Hard Winter Wheat Genetics Research Unit, Agricultural Research Service, USDA, Manhattan, KS, United States; ^5^ Department of Plant Pathology, Kansas State University, Manhattan, KS, United States

**Keywords:** *Fusarium graminearum*, transgenic wheat plants, trichothecene, deoxynivalenol, acetyltransferase, Fusarium head blight

## Abstract

*Fusarium graminearum*, the causal agent of Fusarium head blight (FHB), produces various mycotoxins that contaminate wheat grains and cause profound health problems in humans and animals. Deoxynivalenol (DON) is the most common trichothecene found in contaminated grains. Our previous study showed that Arabidopsis-expressing *F. graminearum* trichothecene 3-*O*-acetyltransferase (*FgTRI101*) converted DON to 3-acetyldeoxynivalenol (3-ADON) and excreted it outside of Arabidopsis cells. To determine if wheat can convert and excrete 3-ADON and reduce FHB and DON contamination, *FgTRI101* was cloned and introduced into wheat cv Bobwhite. Four independent transgenic lines containing *FgTRI101* were identified. Gene expression studies showed that *FgTRI101* was highly expressed in wheat leaf and spike tissues in the transgenic line FgTri101-1606. The seedlings of two FgTri101 transgenic wheat lines (FgTri101-1606 and 1651) grew significantly longer roots than the controls on media containing 5 µg/mL DON; however, the 3-ADON conversion and excretion was detected inconsistently in the seedlings of FgTri101-1606. Further analyses did not detect 3-ADON or other possible DON-related products in FgTri101-1606 seedlings after adding deuterium-labeled DON into the growth media. FgTri101-transgenic wheat plants showed significantly enhanced FHB resistance and lower DON content after they were infected with *F. graminearum*, but 3-ADON was not detected. Our study suggests that it is promising to utilize *FgTRI101*, a gene that the fungus uses for self-protection, for managing FHB and mycotoxin in wheat production.

## Introduction

Fusarium head blight (FHB) caused by *Fusarium* species, especially *Fusarium graminearum*, is a devastating disease of wheat and barley worldwide. FHB not only results in crop yield losses but also contaminates grains with a variety of trichothecene mycotoxins. Deoxynivalenol (DON) is the most common foodborne mycotoxin found in contaminated grains and derived products and poses a threat to food safety and security (Pestka, 2010). Current control strategies for FHB include growing resistant cultivars, fungicide application, and biocontrol. For genetic resistance, hundreds of quantitative traits loci (QTLs) related to FHB resistance have been reported in wheat and its distant relatives, but only a few of them show a major effect on FHB resistance and have been used in breeding, including *Fhb1*, *Fhb5*, and *Fhb7* (Buerstmayr et al., 2020; Wang et al., 2020). Extensive application of fungicides has resulted in fungicide-resistant *F. graminearum* strains (Spolti et al., 2014). A recent study found that a single amino acid change (G443S) in the *CYP51A* gene of *F. graminearum* greatly reduced fungicide sensitivity (Zhao et al., 2022). Therefore, alternative methods are urgently needed to control FHB and mycotoxin contamination.

During the interaction between *Fusarium* and wheat, DON acts as a virulence factor to facilitate the pathogen pass through rachis nodes and spread to neighboring spikelets (Proctor et al., 1995). Multiple effectors have been identified that suppress plant immunity and promote FHB development (Hao et al., 2019, Hao et al., 2020). In addition, FHB severity and mycotoxin contamination are greatly affected by environmental factors such as temperature and carbon dioxide (Hay et al., 2021), which pose challenges to management of FHB and mycotoxin contamination.

In *F. graminearum*, trichothecene biosynthetic genes (*TRI*) have been well characterized genetically and functionally. One key *TRI* cluster contains 12 *TRI* genes in chromosome 2. The trichothecene-producing *Fusarium* species contain a *TRI101* gene encoding trichothecene 3-*O-* acetyltransferase, which converts isotrichodermol to isotrichodermin by adding an acetyl group at position 3 (McCormick et al., 1999). Acetylation of DON to 3-ADON is considered a detoxification mechanism for fungal self-protection. *Saccharomyces cerevisiae* expressing *FgTRI101* can grow in the presence of threefold to fivefold higher levels of DON than yeast without the gene (Kimura et al., 1998). DON is known to interact with eukaryotic ribosomes to inhibit protein biosynthesis. Ribotoxin studies of molecular docking revealed that modifications at the position 3 of DON significantly impaired the ability to interact with ribosomal binding sites (Dellafiora et al., 2017).

It is appealing to utilize Tri101 to detoxify trichothecenes and reduce toxin contamination in wheat and barley. Prior studies have attempted to express *Tri101* from *F. sporotrichioides* (*FsTRI101*) in plants to reduce FHB primarily caused by *F. graminearum*. The transgenic tobacco expressing *FsTRI101* showed significantly increased tolerance to the trichothecene 4,15-diacetoxyscirpenol (DAS), a substrate of FsTri101 (Muhitch et al., 2000). However, the transgenic wheat expressing FsTri101 did not significantly increase FHB resistance (Okubara et al., 2002). Although the acetyltransferase activity of FsTri101 was detected in one of the four lines expressing *FsTRI101*, no conversion of DON to 3-ADON was reported in the transgenic line (Okubara et al., 2002). Subsequently, comparison of FsTri101 and FgTri101 protein structures and activities have demonstrated that FgTri101 is 80-fold more effective at binding DON than FsTri101 (Garvey et al., 2008). Our recent study showed that the transgenic Arabidopsis expressing *FgTRI101* converted DON to 3-ADON and excreted 3- ADON out of Arabidopsis cells (Hao et al., 2021).

To examine if transgenic wheat-expressing *FgTRI101* can convert and excrete DON as Tri101 transgenic Arabidopsis does, and provide enhanced FHB resistance, in this study, we cloned *FgTRI101* and generated transgenic wheat expressing *FgTRI101*. We examined whether the transgenic wheat seedlings expressing the *FgTRI101* could acetylate DON and excrete 3-ADON. Furthermore, we conducted FHB virulence assays and determined whether *FgTRI101* transgenic wheat plants reduce FHB and mycotoxin contamination.

## Materials and methods

### Plasmid construction

*Fusarium graminearum TRI101* gene was amplified from a plasmid pBinARS/plus-*FgTRI101* constructed for Arabidopsis transformation (Hao et al., 2021). The primer sets, *FgTRI101*-BamHIF and *FgTRI101*-BamHIR, were used and included *Bam*HI sites (underlined) for cloning. The amplified DNA fragment was digested, purified, and ligated into pAHC17 vector at the *Bam*HI site (Christensen and Quail, 1996). The orientation and sequence of the inserted fragment in the construct was confirmed by sequencing using an ABI 3730 DNA analyzer (Applied Biosystems, Waltham, MA, USA). The expression constructs contained the *FgTRI101* gene driven by a maize ubiquitin promoter (Ubi-1) and terminated with a nopaline synthase terminator (Nos-t).

### Wheat transformation and regeneration

Immature embryos were isolated from spring wheat (*Triticum aestivum* L.) cv. Bobwhite grown in a controlled environment with a 16h photoperiod, and the day/night temperatures at 20/18°C. The *FgTRI101* expression construct and the pAHC20 (Christensen and Quail, 1996) containing the bar gene were co-bombarded into selected wheat embryogenic calli with a 1:1 ratio. Biolistic transformation was conducted with a particle inflow gun and then tissue culture and plant regeneration were performed (Tian et al., 2019). Leaves of recovered plants grown in soil were screened for herbicide resistance by brushing a 0.2% v/v Liberty (glufosinate) solution (AgroEvo USA, Wilmington, DE). The putative herbicide-resistant plants without necrosis after 5 days of Liberty application were analyzed by polymerase chain reaction (PCR) for the presence of the *FgTRI101* using primers FgTRI101-BamH1F and FgTRI101-BamH1F (**Supplementary Table S1**). The transgenic plants (T_2_) carrying *FgTRI101* were selected for FHB evaluation.

### Molecular analysis of transgenic wheat

Plant genomic DNA was isolated from leaves of 7- to 10-day-old transgenic seedlings using ZR Fungal/Bacterial DNA Miniprep Kit (Zymo Research, Boston, MA). DNA was quantified using a spectrophotometer (NanoDrop 2000, Thermo Fisher Scientific, Waltham, MA). PCR was used to amplify the target gene *FgTRI101* using the primers FgTRI101-BamHIF and FgTRI101-BamHIR (**Supplementary Table S1**).

Total RNA was extracted from leaves and spikes of *FgTRI101*-positive transgenic wheat plants using Trizol reagent (Sigma-Aldrich, St. Louis, MO) combined with the Ambion RNA isolation and RQ1 RNase-free DNase treatment kit (Thermo Fisher Scientific, Waltham, MA). RNA was quantified with a spectrophotometer (Nanodrop; Thermo Fisher Scientific, Waltham, MA) and verified for the absence of genomic DNA contamination by real-time quantitative PCR (qPCR). The first-strand cDNA was synthesized, and reverse transcriptase qPCR (RT-qPCR) was performed on a Bio-Rad CFX96 RealTime System (Bio-Rad Laboratoriesm, Hercules, CA, USA) using primers FgTRI101-RT-F and FgTRI101-RT-R (**Supplementary Table S1**). *FgTRI101*-expression level was calculated with the 2^-ΔΔCt^ method using CFX Manager software (Bio-Rad), relative to the transgenic line with the lowest expression level (Tri101-2128), which was set as a value of 1. The wheat gene glyceraldehyde 3-phosphate dehydrogenase (*GAPDH*) was used to normalize the values as an internal control. The qPCR reactions were performed in triplicate and repeated three times.

To obtain homozygotes for further analysis, T_1_ seeds from *FgTRI101*-positive transgenic lines were planted. DNA was isolated from leaves of T_1_ seedlings using ZR Fungal/Bacterial DNA miniprep kit (Zymo Research, Boston, MA, USA). The positive seedlings were determined by PCR screening. Furthermore, *FgTRI101* copy numbers in the *FgTRI101-*positive seedlings were estimated by qPCR as described (Hao et al., 2021). Wheat gene *Epsilon Cyclase* (*TaEC*), which is a single-copy gene, was used as the endogenous reference gene (Mieog et al., 2013). Primer efficiency for *FgTRI101* and *TaEC* was determined using a standard curve with DNA dilution series. The qPCR amplification efficiency was calculated according to the following equation: Efficiency = 10 ^(−1/slope)^ − 1. The amplification efficiency of the target gene *FgTRI101* was 99.5% and wheat TaEC is 105%. The ratio of the copy number of *TRI101* was determined using the following equation: Ratio = [1 + Efficiency (Ct*_FgTri101_*)]/[1 + Efficiency (Ct*_TaEC_*)]. The qPCR reactions were set up in three technical triplicates and repeated three times. Data are shown as means ± SD of three replicates.

### Mycotoxin assays

15-ADON was isolated from a *F. graminearum* mutant strain B4-1 (Chen et al., 2000); DON was made by hydrolysis of 15-ADON with 0.1 N sodium hydroxide. A stock solution of DON (2 mg/mL in water) was prepared for feeding experiments. Deuterium-labeled DON (1 mg/mL solution) was purchased from Sigma-Aldrich (St. Louis, MO).

### Trichothecene sensitivity assays using wheat seedlings

T_2_ wheat seeds of the transgenic plants expressing *FgTRI101* (lines 1451, 1606, 1651, and 2128) and control line BW1410 were surface sterilized using 70% ethanol for 2 min, then 50% Clorox for 10 min, and rinsed three times with sterile water. The seeds were placed on Murashige and Skoog (MS, Sigma-Aldrich, St. Louis, MO) media containing 5 µg/mL of DON in vertical plates (Greiner bio-one North America Inc., Monroe, NC). A total of ten seeds were placed on each agar plate with three replications for each transgenic line. Root growth was measured at 15 days after planting. Means of root length were compared using JMP and Dunnett’s method (*n* = 10–18). The experiments were conducted at least three times with similar results.

### Detection of DON and 3-ADON in transgenic wheat seedlings

T_2_ seeds of the wheat transgenic plants expressing *FgTRI101* (Tri101-1451, -1606, -1651, and -2128) and control BW1410 were sterilized as described above and used for DON acetylation assays (Hao et al., 2021). The sterilized seeds were placed on agar plates and maintained in a growth chamber at 23/20°C with a 16/8h light/dark cycle for germination. After 3 days, six seedlings were transferred into one 50-mL tube containing 5 or 10 mL of half-strength MS liquid medium with 1% sucrose and 50 µg/mL DON. Three tubes were used for each treatment. After 7 days, the samples were collected, weighed and then ground in liquid nitrogen. The ground seedling samples and culture media were measured for toxin content by GC/MS. Media containing toxins without plants and *FgTRI101*-negative BW1410 served as controls. The experiments were conducted at least eight times. Similarly, 3-ADON and deuterium-labeled DON were used to test 3-ADON stability in wheat seedlings.

### Mycotoxin extraction and quantification

Wheat tissues were extracted using 10 mL of acetonitrile: water (86:14) by shaking for 1h at 250 rpm on an orbital shaker. The samples were centrifuged for 5 min at 4,000 rpm, and 9 mL of supernatant was dried, derivatized, and analyzed as described (Hao et al., 2019). To measure toxins in liquid media, 1.4 mL aliquots of media were added to 8.6 mL of acetonitrile and the mixture was dried under a stream of air. Toxins were derivatized and analyzed by GC/MS as described (Hao et al., 2019).

### FHB virulence assays

FgTri101-transgenic plants and control plants were cultivated in a growth chamber. Each transgenic line was planted in four pots, with five seeds sown in each pot containing SunShine Mix (Sun Gro Horticulture, Agawam, MA) with addition of 100 g Osmocote and 15 g Micromax in 5 L soil. The pots were randomly placed in the growth chamber. The growth chamber was set with 16h light at 23°C and 8h dark at 20°C with 50% relative humidity. Wheat plants were watered as needed and fertilized every two weeks with a solution containing 325 mg/L of Peter’s 20:20:20 (Grace-Sierra Horticultural Products, Milpitas, CA) until inoculation.

To determine if *FgTRI101* expressing wheat plants reduce FHB severity and DON contamination, FHB virulence assays were performed (Hao et al., 2019). Briefly, macroconidia of *F. graminearum* strain PH-1 were collected from 4-day-old mung bean liquid cultures. The inoculum was filtered, centrifuged, and adjusted to a concentration of 10^5^ conidia/mL. At mid-anthesis, 10 µL of conidia solution was placed between paleae and lemma of the fifth floret from the top of a spike. Eighteen or twelve spikes were inoculated for each transgenic line. Each inoculated head was considered as a replicate. Inoculated wheat spikes were covered with a plastic bag for 3 days to maintain high humidity. FHB progress was evaluated at 14 days post-inoculation (dpi). At 14 dpi, individual wheat spikes were harvested. For each transgenic line, two or three inoculated wheat spikes were combined, lyophilized, and pulverized in 2.5 oz silver aluminum seamless screw top can (Freund Container & Supply, Lisle, IL) with four to five 3/8-in. diameter stainless steel grinding balls (SPEX SamplePrep, Metuchen, NJ). The pulverized tissues were measured for DON and 3-ADON content using GC/MS. Statistical analysis was performed with one way analysis of variance using JMP (*n* = 12–18).

## Results

### Transformation of *FgTRI101* into wheat cultivar Bobwhite

**Figure 1:**
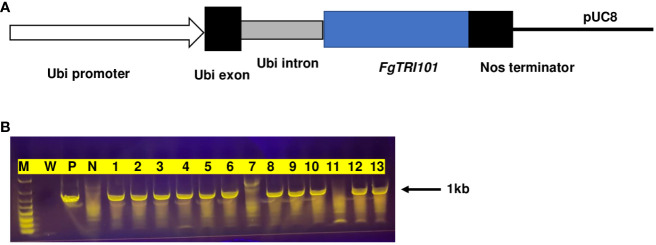
Generation of *FgTRI101* transgenic wheat lines. **(A)** A diagram of the construct showing the 2.6-kb maize Ubi-1 promoter (arrow), exon (black) and intron (gray), the 1.4-kb *FgTRI101* coding region (blue), the NOS terminator (black) and pUC8 vector (black line). **(B)** The presence(+)/absence(−) of the *FgTRI101* fragment in the representative T_1_ seedlings was analyzed by PCR using primers FgTRI101-ORF-F and FgTRI101-ORF-R. M, molecular marker; W, water as a negative control; P, positive control amplified from plasmid. N, Bobwhite (BW1410) as a negative control. Lanes 1–13: plants from transgenic lines FgTri101-1451 (lanes 1–3), FgTri101-1606 (lanes 4–6), FgTri101-1651 (lanes 7–9), and FgTri101-2128 (lanes 10–13). Lanes 7 and 11 are absent of *FgTRI101* gene.

*FgTRI101* driven by Ubi-1 was cloned and transformed into wheat cv. Bobwhite. After transformation and selection, four independent transgenic plants containing the *FgTRI101* gene, designated as FgTri101-1451, -1606, -1651, and -2128, were obtained after PCR screening (**Figure 1**). A total of 47 T_1_ seedlings from the four transgenic lines were screened and 24 positive seedlings were identified and examined for copy numbers by qPCR (**Supplementary Table S2**). The transgenic plants containing a single copy of *FgTRI101* (FgTri101-1451-3, -1606-10, -1651-3, and -2128-3) were selected from the positive transgenic lines and one transgenic line lacking *FgTRI101* (BW1410) was selected as a negative control for further functional analyses.

**Figure 2:**
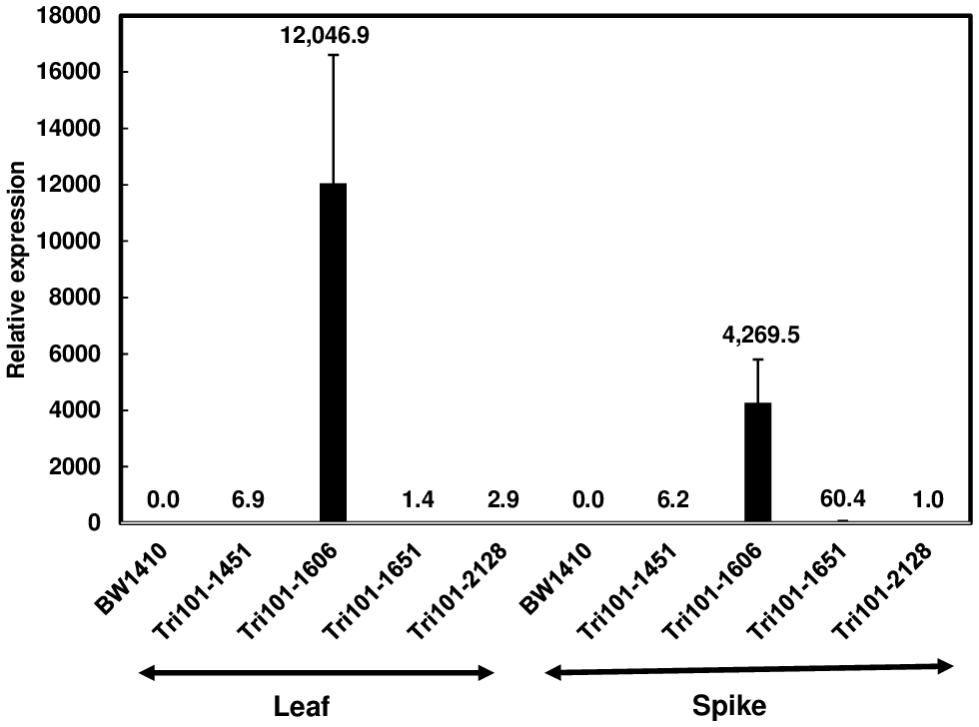
*FgTRI101* expression in the wheat transgenic plants. The expression of *FgTRI101* was normalized to the expression of wheat gene (*TaGAPDH*). The relative gene expression was calculated using the 2^-ΔΔCt^ method. A sample value was expressed versus FgTri101-2128 expression in spikes, which had the lowest expression among the tested samples. The experiments were conducted with three technical and three biological replicates. Bars represent the means from three biological replicates and standard deviations.

RT-qPCR was used to evaluate the expression of *FgTRI101* in the transgenic lines. The expression of *FgTRI101* was high in line FgTri101-1606, but relatively low in the other three lines (**Figure 2**). *F. graminearum* infects wheat spikes to cause FHB and previous studies demonstrated that Ubi-1 promoter is active in endosperm (Stoger et al., 1999); therefore, we also examined *FgTRI101* expression in wheat spikes at mid-anthesis. Spikes of the FgTri101-1606 line displayed the highest expression level although the expression level was lower in spikes than in leaves (**Figure 2**). In contrast, the expression of *FgTRI101* was higher in spikes than in leaves in the FgTri101-1651 line. Both FgTri101-1451 and -2128 lines had relatively low levels of *FgTRI101* expression in leaves and spikes (**Figure 2**).

### Roots of *FgTri101* transgenic wheat enhanced DON resistance

**Figure 3:**
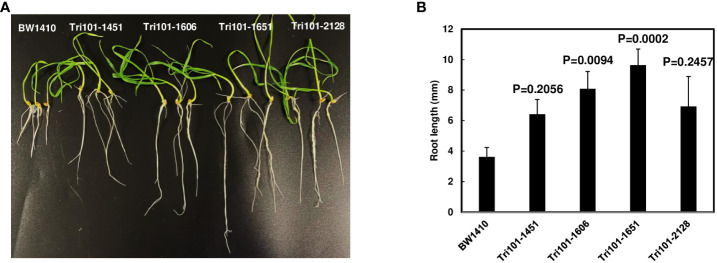
Wheat seedling growth on MS media containing trichothecene DON (5 µg/mL). Four transgenic lines (FgTri101-1451, -1651, -1606, and -2128) and a control transgenic line BW1410 were used. **(A)** Phenotypes. The photographs were taken after a 2-week incubation; **(B)** Comparison of the root length, which were measured at 15 days. Means of root length were compared by one way ANOVA and Dunnett’s method using JMP (*n* = 10–18). Different letters indicate significant differences. The experiments were conducted three times with similar results.

Our prior studies showed *Arabidopsis* expressing *FgTRI101* germinated and grew well on MS medium with addition of 10 µg/mL DON (Hao et al., 2021). First, we examined if wheat expressing *FgTRI101* increased DON resistance as *Arabidopsis* expressing *FgTRI101* did. In our preliminary study, none of transgenic wheat lines expressing *FgTRI101* or the control line germinated on MS medium containing 10 µg/mL DON. Then, the ability of transgenic wheat root growth on MS medium containing 5 µg/mL DON was evaluated. On average, 50% seeds germinated on MS medium containing 5 µg/mL DON. Germinated and ungerminated BW1410 seeds had necrotic and rotten symptoms on seed coats, whereas the germinated and ungerminated transgenic lines remained healthy. The germination rate of line Tri101-2128 was significantly lower than the other three lines. At 15 days after sowing, wheat root lengths were measured and compared. Significantly longer roots were observed in transgenic lines FgTri101-1651 (*p* = 0.0002) and FgTri101-1606 (*p* = 0.0094) compared to the control line (**Figure 3**). This result indicates that FgTri101 transgenic wheat seedlings have greater tolerance to DON than the control wheat line.

### Conversion of DON to 3-ADON in *FgTRI101* expressing transgenic wheat

**Figure 4:**
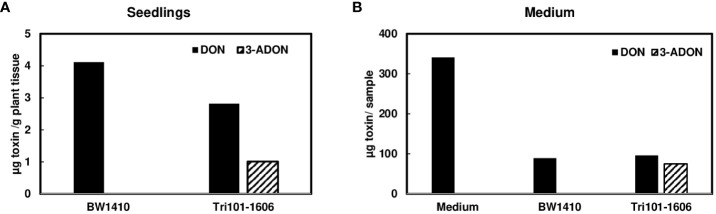
Conversion of deoxynivalenol (DON) to 3-ADON by FgTri101 transgenic wheat seedlings. **(A)** DON and 3-ADON extracted from wheat seedlings after treatment with 50 µg/mL of DON for 7 days. All plant tissues were extracted for toxins; **(B)** DON and 3-ADON extracted from media after wheat seedlings treated with 50 µg/mL of DON for 7 days. Media without wheat seedlings served as a control. Toxins in 1.5 mL aliquots from 10 mL media was measured. Total toxin in liquid was presented by formula: toxin/1.5 × 10. The experiments were repeated at least 8 times. This was one of two experiments showing of DON to 3-ADON conversion and excretion.

Although we observed that *Tri101* transgenic Arabidopsis converted DON to 3-ADON efficiently and excreted 3-ADON to the medium (Hao et al., 2021), the conversion of DON to 3-ADON was not consistently detected in FgTri101 transgenic wheat seedlings or media. With multiple attempts, we only detected low levels of DON to 3-ADON conversion occasionally when DON was added to the media culturing transgenic line FgTri101-1606 (**Figure 4A**). Once DON was converted to 3-ADON, 3-ADON was excreted into the medium (**Figure 4B**). To determine if 3-ADON is stable in monocot plants, we added 50 µg/mL 3-ADON to the media used to culture wheat seedlings of FgTri101-1606 and BW1410. Surprisingly, both 3-ADON and DON were detected in FgTri101-1606 and BW1410 seedlings but at a relatively low level (**Supplementary Figure S1A**), in contrast, higher amounts of 3-ADON were observed in the media (**Supplementary Figure S1B**). These observations suggest that 3-ADON is stable in wheat seedlings and wheat can excrete 3-ADON.

Next, to test the fate of 3-ADON in seedlings expressing *FgTRI101*, deuterium-labeled DON was added to the media with growing transgenic *FgTri101*-1606 seedlings. Neither labeled 3-ADON nor other potential DON-related products were detected in seedlings or media (data not shown). Taken together, these data indicate that *Tri101* transgenic wheat can convert DON to 3-ADON, but the fate of 3-ADON is unclear.

### Transgenic wheat expressing *FgTRI101* enhances FHB resistance and reduces DON contamination

**Figure 5:**
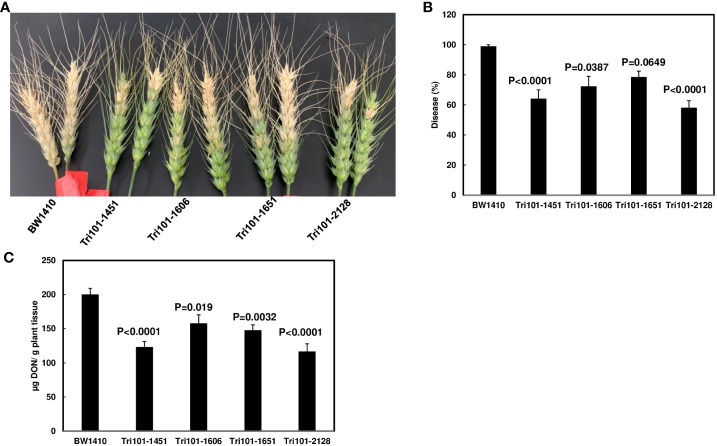
Reduction of Fusarium head blight (FHB) and deoxynivalenol (DON) in transgenic wheat plants expressing *FgTRI101*. Point inoculations (10 µL of spore suspension containing 1,000 conidia) were performed on wheat florets (cv. Bobwhite) with *F*. *graminearum* wild-type PH-1. **(A)** The positive transgenic lines (FgTri101-1451, 1606, 1651, and 2128) displayed higher level of resistance to FHB compared to the control transgenic line (BW1410). Images were taken at 14 days post inoculation (14 dpi). **(B)** FHB severity. FHB was scored at 14 dpi and calculated as percentage of symptomatic spikelets per spike. **(C)** DON content. Infected heads were collected at 14 dpi for analysis. Statistical analyses for **(B, C)** were performed with one way ANOVA and Dunnett’s method using JMP (*n* = 18 for FHB severity and *n* = 6 for DON). Different letters indicate significant differences.

FHB resistance was evaluated by single floret inoculation and disease symptoms were scored at 14 dpi. The negative control line BW1410 exhibited almost 100% symptomatic spikelets at 14 dpi, whereas the transgenic lines (FgTri101-1451 and 2128) showed significantly lower disease score (**Figure 5**, *p* < 0.05). Furthermore, toxin analysis showed significantly lower DON levels in all four transgenic lines than in the control; however, 3-ADON was not detected in the infected tissues. These data indicate that *FgTRI101* reduces DON levels, which led to less FHB severity.

### Morphology of transgenic wheat plants expressing *FgTRI101*

To assess whether transgenic wheat expressing *FgTRI101* changes wheat growth rate, morphology, or agronomic traits, all four *FgTRI101* transgenic lines and the control BW1410 were planted in a growth chamber to compare their flowering time, plant height, floret number per spike, and seed weight per 100 seeds. The *FgTRI101* lines were similar to the control BW1410 for flowering time, floret number and seed weight. In contrast, all four *FgTRI101* lines had significantly taller plants than BW1410 control (**Table 1**). Nevertheless, the expression of the *FgTRI101* in wheat did not show any significant negative effects on those agronomic characters.

**Table 1:** Comparison of morphological traits among transgenic wheat plants expressing *FgTRI101* and control.

Genotypes	Flower time (day)	Florets/spike^a^	Plant height (cm)^b^	Weight/100 seeds
BW1410	48	17	48.68b	3.81
Tri101-1451	44	17	52.82a	3.92
Tri101-1606	48	19	52.38a	3.81
Tri101-1651	48	18	54.38a	3.82
Tri101-2128	44	17	53.63a	3.89

^a^Number of florets was counted at 51 days after plants were transferred to soil. ^b^Plant heights were measured when plants were completely dried. Different letters indicate significance at the 0.05 level by one-way ANOVA and Tukey-Kramer HSD.

## Discussion

In this study, we expressed *FgTRI101* in wheat using gene transformation and found significantly increased FHB resistance and less DON accumulation in FgTri101-transgenic wheat. We observed that the root lengths of FgTri101 transgenic wheat seedlings were significantly longer than those of the controls on MS medium containing DON. However, compared to the Arabidopsis expressing *FgTRI101*, FgTri101-transgenic wheat seedlings had less DON tolerance (5 vs. 10 µg/mL). All FgTri101 transgenic Arabidopsis seeds germinated and grew well on MS media supplemented with 10 µg/mL DON (Hao et al., 2021). However, none of the FgTri101 transgenic wheat seeds germinated on MS media supplemented with 10 µg/mL DON. Nevertheless, FgTri101 transgenic wheat seedlings had longer roots than the control on MS media supplemented with 5 µg/mL DON (**Figure 3**). This may be partially due to the different promoters used in generating transgenic Arabidopsis and wheat. The *FgTRI101* gene in transgenic Arabidopsis plants was driven by a double CaMV35S promoter, whereas the expression of *FgTRI101* in transgenic wheat was controlled by a maize Ubi-1 promoter. Although the maize Ubi-1 promoter has been found to be more active in the endosperm (Stoger et al., 1999), we observed relatively higher *FgTRI101* expression in wheat leaves than wheat spikes in the transgenic line FgTri101-1606. In addition, it is also possible that Arabidopsis and wheat differ in their uptake and metabolism of trichothecenes. Previous studies identified an Arabidopsis UDP-glucosyltransferase that effectively converted DON to DON-3-O-glucoside to increase DON resistance (Poppenberger et al., 2003).

Gene expression studies showed varying levels of *FgTRI101* expression in the different transgenic wheat lines. *FgTRI101* expression was high in the transgenic line FgTri101-1606 but was relatively low in the other three lines carrying *FgTRI101* (**Figure 2**). Interestingly, the seedlings of both FgTri101-1606 and FgTri101-1651 lines had significantly increased DON resistance (**Figure 3**). All four FgTri101 transgenic lines had enhanced FHB resistance and reduced DON contamination (**Figure 5**). Some levels of correlation have been observed between the transgene expression and disease resistance (Lu et al., 2013). However, some transgenic studies have shown that transgene expression levels are not always correlated to the levels of disease resistance (Hao et al., 2016). Our results showed that both high and low *FgTRI101* expression in transgenic wheat lines provided DON and FHB resistance as well as toxin reduction. Studies showed a positive correlation between the lactoferrin protein expression levels and the levels of FHB in transgenic wheat (Han et al., 2012). We speculate that FgTri101 protein levels might be better correlated with FHB resistance. Further investigations are needed to produce FgTri101 antibody to quantify its expression at the protein level.

It has been demonstrated that Arabidopsis expressing *FgTRI101* efficiently acetylated trichothecenes and efficiently excreted them out of Arabidopsis cells (Hao et al., 2021). However, in the current study, the detection of 3-ADON in transgenic wheat experiments was inconsistent. When 3-ADON (50 µg/mL) was added to the media containing wheat seedlings, both 3-ADON and DON were detected in plants and media (**Supplementary Figure S1**), indicating that 3-ADON is stable in wheat. Similarly, none of the previous studies detected 3-ADON in transgenic wheat, barley or rice expressing *FsTRI101* or *FgTRI101* (Okubara et al., 2002; Manoharan et al., 2006; Ohsato et al., 2007). It has been suggested that the C-3 acetyl is unstable in plant cells and plant genes in transgenic plants expressing *TRI101* could consistently remove 3-ADON (Ohsato et al., 2007). To support this hypothesis, a previous study found that a carboxylesterase from *Brachypodium distachyon* efficiently deacetylated 3-ADON or 15-ADON to DON and multiple homologs of the carboxylesterase were identified in wheat (Schmeitzl et al., 2016). For instance, when wheat cell suspension culture was supplemented with 75 µg/mL 3-ADON, almost all 3‐ADON was converted to DON and transported out of the cells, and only 4% 3‐ADON was detected in the supernatant after 96 h (Schmeitzl et al., 2015). In the current study, DON was detected in wheat seedlings when supplemented with 3-ADON (**Supplementary Figure S1**), suggesting that at least one functional carboxylesterase is present in wheat. Taken together, it is possible that there is a limited amount of 3-ADON converted from DON by *FgTRI101* in the transgenic plants, but it might be rapidly deacylated to DON by carboxylesterases. In *FgTRI101*-expressing heads inoculated with *F. graminearum*, we speculate that the transformation between DON and 3-ADON is continuously ongoing. Although DON is converted to 3-ADON by *FgTRI101*, 3-ADON is converted to DON by wheat carboxylesterases. DON is known to act as a translational inhibitor in eukaryotic cells by inhibiting 60S ribosomal subunits. In contrast, 3-ADON is almost nontoxic to the ribosomal target. Using *in-silico* analysis, a recent study found that steric hindrances in the position 3 of the trichothecene core may result in the loss of ribotoxicity (Dellafiora et al., 2017). Since 3-ADON is less toxic to plants than DON and it might cause less damage to infected wheat heads, 3-ADON may play less of a role in FHB spread. Further studies are needed to confirm this hypothesis. Nevertheless, our data showed significantly reduced DON in FgTri101 transgenic spikes compared to control spikes after infection with *F. graminearum* strain PH-1, which lead to reduced FHB.

In conclusion, we expressed *FgTRI101* in transgenic wheat and demonstrated that transgenic wheat seedlings had increased DON resistance. Furthermore, we showed that *FgTRI101* transgenic wheat reduced FHB severity and DON levels. Therefore, it is promising to utilize *FgTRI101* control FHB and DON contamination. To enhance *FgTRI101* protein expression level and FHB resistance, *FgTRI101* will be wheat-codon optimized and introduced to FHB tolerant and commercial varieties used in the field.

## Data availability statement

The datasets presented in this study can be found in online repositories. The names of the repository/repositories and accession number(s) can be found below: https://www.ncbi.nlm.nih.gov/genbank/, XM_011329426.

## Author contributions

GY-S: Investigation, Methodology, Writing – review & editing. SM: Conceptualization, Data curation, Investigation, Methodology, Writing – review & editing. HC: Data curation, Investigation, Methodology, Writing – review & editing. GB: Funding acquisition, Writing – review & editing. HT: Funding acquisition, Investigation, Methodology, Writing – review & editing. GH: Conceptualization, Data curation, Formal Analysis, Funding acquisition, Investigation, Methodology, Project administration, Supervision, Validation, Writing – original draft, Writing – review & editing.
