# Supplementary material for: Reduction of Fusarium head blight and trichothecene contamination in transgenic wheat expressing Fusarium graminearum trichothecene 3-O-acetyltransferase
Source: Front Plant Sci. 2024 Apr 8;15:1389605. doi: 10.3389/fpls.2024.1389605 (PMC11033581; doi:10.3389/fpls.2024.1389605)
Supplement: Supplementary file 1 [file DataSheet_1.docx]

**Supplementary**

**Fig. S1.** Detection of DON and 3-ADON in wheat seedlings FgTri101-1606 and BW1410 when supplemented with 3-ADON. **A,** DON and 3-ADON in wheat seedlings after treatment with 50 µg/mL 3-ADON for 7 days. All plant tissues were extracted for toxins; **B,** DON and 3-ADON extracted from media after wheat seedlings treated with 50 µg/mL DON for 7 days. Media without wheat seedlings served as a control. Toxins in 1.5 mL aliquots from 5 mL media was measured and total toxin in liquid was presented by formula: toxin/1.5×5.





**Table S1 Primers used in this study**

|  |  |
| --- | --- |
| **Gene name** | **Primer sequence** |
| FgTRI101-BamHIF | 5'-AGTGGATTCATGGCTTTTCAAGATACAGCTC-3' |
| FgTRI101-BamHIR | 5'-GAGAGGATTCTAACCGTACTGCGCATA-3' |
| FgTRI101-RT-F | 5'-GATCTACACCCAAATCAGTCTCC-3' |
| FgTRI101-RT-R | 5'-GCTCGAAGGTGCTGACAATAg-3' |
| TaGAPDH-F | 5'-TTGCTCTGAACGACCATTTC-3' |
| TaGAPDH-R | 5'-GACACCATCCACATTTATTCTTC-3' |
| TaEC-A-RT-F | 5'-ACCTGACCTTGTAAAACCATTCAT-3' |
| TaEC-A-RT-R | 5'-TGACATCCTCCAACATCTCTAAC-3' |
|  |  |

**Table S2 *FgTRI101* copy numbers in transgenic wheat**

|  |  |  |
| --- | --- | --- |
| Transgenic line | Ratio ± SD | Estimated *TRI101* copy number |
| FgTri101-T1-1451-1 | 1.06±0.04 | 1 |
| FgTri101-T1-1451-2 | 1.52±0.31 | 2 |
| FgTri101-T1-1451-3 | 1.44±0.18 | 1 |
| FgTri101-T1-1451-7 | 0.99±0.27 | 1 |
| FgTri101-T1-1451-11 | 0.69±0.01 | 1 |
| FgTri101-T1-1451-12 | 0.74±0.02 | 1 |
| FgTri101-T1-1451-16 | 0.91±0.20 | 1 |
| FgTri101-T1-1606-2 | 0.99±0.07 | 1 |
| FgTri101-T1-1606-3 | 0.83±0.08 | 1 |
| FgTri101-T1-1606-5 | 0.73±0.08 | 1 |
| FgTri101-T1-1606-8 | 0.83±0.00 | 1 |
| FgTri101-T1-1606-9 | 0.82±0.03 | 1 |
| FgTri101-T1-1651-1 | 0.84±0.04 | 1 |
| FgTri101-T1-1651-3 | 0.93±0.31 | 1 |
| FgTri101-T1-1651-4 | 0.85±0.18 | 1 |
| FgTri101-T1-1651-5 | 0.87±0.27 | 1 |
| FgTri101-T1-1651-6 | 0.81±0.01 | 1 |
| FgTri101-T1-1651-8 | 0.85±0.02 | 1 |
| FgTri101-T1-1651-9 | 1.10±0.20 | 1 |
| FgTri101-T1-1651-10 | 0.84±0.07 | 1 |
| FgTri101-T1-1651-11 | 0.83±0.08 | 1 |
| FgTri101-T1-2128-2 | 0.85±0.08 | 1 |
| FgTri101-T1-2128-3 | 0.74±0.00 | 1 |
| FgTri101-T1-2128-4 | 0.76±0.03 | 1 |
|  |  |  |
|  |  |  |
